# Population Recovery following Decline in an Endangered Stream-Breeding Frog (*Mixophyes fleayi*) from Subtropical Australia

**DOI:** 10.1371/journal.pone.0058559

**Published:** 2013-03-13

**Authors:** David Alan Newell, Ross Lindsay Goldingay, Lyndon Owen Brooks

**Affiliations:** 1 School of Environment, Science and Engineering, Southern Cross University, New South Wales, Australia; 2 Marine Ecology Research Centre, Southern Cross University, New South Wales, Australia; Smithsonian's National Zoological Park, United States of America

## Abstract

Amphibians have undergone dramatic declines and extinctions worldwide. Prominent among these have been the stream-breeding frogs in the rainforests of eastern Australia. The amphibian chytrid fungus *Batrachochytrium dendrobatidis* (*Bd*) has been postulated as the primary cause of these declines. We conducted a capture-mark-recapture study over a 7-year period on the endangered Fleay’s barred frog (*Mixophyes fleayi*) at two independent streams (30 km apart) in order to assess the stability of these populations. This species had undergone a severe decline across its narrow geographic range. Mark-recapture modelling showed that the number of individuals increased 3–10 fold along stream transects over this period. Frog detection probabilities were frequently above 50% but declined as the populations increased. Adult survival was important to overall population persistence in light of low recruitment events, suggesting that longevity may be a key factor in this recovery. One male and female were present in the capture record for >6 years. This study provides an unambiguous example of population recovery in the presence of *Bd.*

## Introduction

Amphibians are now acknowledged as the world’s most extinction-prone vertebrates, with 33% of currently described species recognised as threatened [1]. This estimate is conservative because there are insufficient data to adequately assess the status of almost one quarter of all species. In Australia, about one quarter (23%) of known species (216) is considered to be of conservation concern [2]. The amphibian chytrid fungus *Batrachochytrium dendrobatidis* (hereafter *Bd*) has been implicated as the cause of the decline in many species [2], [3], [4].

The subtropical and tropical rainforests of eastern Australia are of particular significance to global amphibian declines because more than 10 stream-breeding frog species have declined or disappeared from these ecosystems since 1979 [5], [6], [7], [8]. *Bd* has been detected in dead and moribund frogs from throughout this broad geographic area, and has been shown to directly cause death in some species [3], [9]. As a consequence, this area has become important in the study of *Bd* infection in frogs [10], [11], [12], [13]. This research has revealed that infection is widespread, that infection rates vary substantially over the year, and that populations can persist with endemic infections [10], [11], [14], [15], [16], [17].

Of considerable interest in these rainforests is the discovery of remnant populations of some frog species that had disappeared from broad areas [10], [12], [15], [17], [18]. The reason for these populations persisting is currently unknown but could be due to the presence of microhabitats that provide thermal environments or antifungal agents that reduce infection prevalence [12], [19], [20], [21], due to the absence of *Bd* (but see [10]), or from the rise of resistant individuals as a consequence of natural selection.

A key question that arises is what is the fate of species that previously suffered rapid declines? Do they struggle along at low abundance in some equilibrium with continuing bouts of infection [10], [12], [16], [22] but at continued risk of extirpation, or is it possible that the pathogen could lose its virulence so species can increase to their former levels of abundance? Answering these questions can be complicated by the fact that amphibian populations may undergo natural fluctuations [23]. The need for detailed population studies to distinguish declining amphibian populations from those exhibiting natural fluctuations has been recognised for many years [23], [24]. Thus, studies that span more than just a few years are needed to resolve this.

We address the question of the fate of a species that underwent a decline using Fleay’s barred frog (*Mixophyes fleayi*). This species provides an example of a stream-breeding frog from Australia’s subtropical rainforests that underwent population declines across its range. *Mixophyes fleayi* is historically known to have a narrow and disjunct distribution from the Conondale Ranges in southeast Queensland south to the Yabbra scrub in northern New South Wales, with an area of occupancy of <500 km^2^. By the mid-late 1990s, this large (60–90 mm SV) frog was absent from many formerly occupied areas whilst remaining populations were characterised by low abundance [8], [25]. It is now known from about 30 scattered locations [8] and is currently listed as endangered by State and Federal legislation. *Bd* is implicated in the decline of this species as it has been in the extinction of two species in the region [4]. *Bd* caused the death of *M. fleayi* at two locations in southeast Qld [3], [8] and has been recorded in *M. fleayi* at other locations where earlier declines had occurred [26], [27]. Furthermore, the congeneric *M. fasciolatus* is highly susceptible to infections of *Bd* [3], [28].

We conducted a mark-recapture study of *M. fleayi* at two disjunct locations over a 7-year period. Our aim was to characterise the trajectory of these two populations of this *Bd*-sensitive species. Abundance was relatively low at both locations at the start of our study [25]. We predict that these populations will show either: i) recurring periods of decline such that abundance at the end of the study period is little different to that at the start or, ii) that over this period abundance increases to be substantially higher than at the start. We have not studied levels of *Bd* infection in these populations. What is central to understanding the medium-term response of these populations is that *Bd* was recorded in both populations through histological examination early in our study (D.A. Newell unpubl. data; M. Mahony unpubl. data). Furthermore, *Bd* has been detected in various species throughout our study region [9], [11], [13], [16], [26], [27]. We are not aware of any evidence that *Bd* has disappeared from any known location in eastern Australia, so it is plausible that both our study populations have on-going exposure to endemic infections of *Bd* (see [10], [13], [16]). Thus, detailed mark-recapture modelling allows us to describe the dynamics of remnant populations of *M. fleayi*.

## Methods

### Study Area

**Figure 1:**
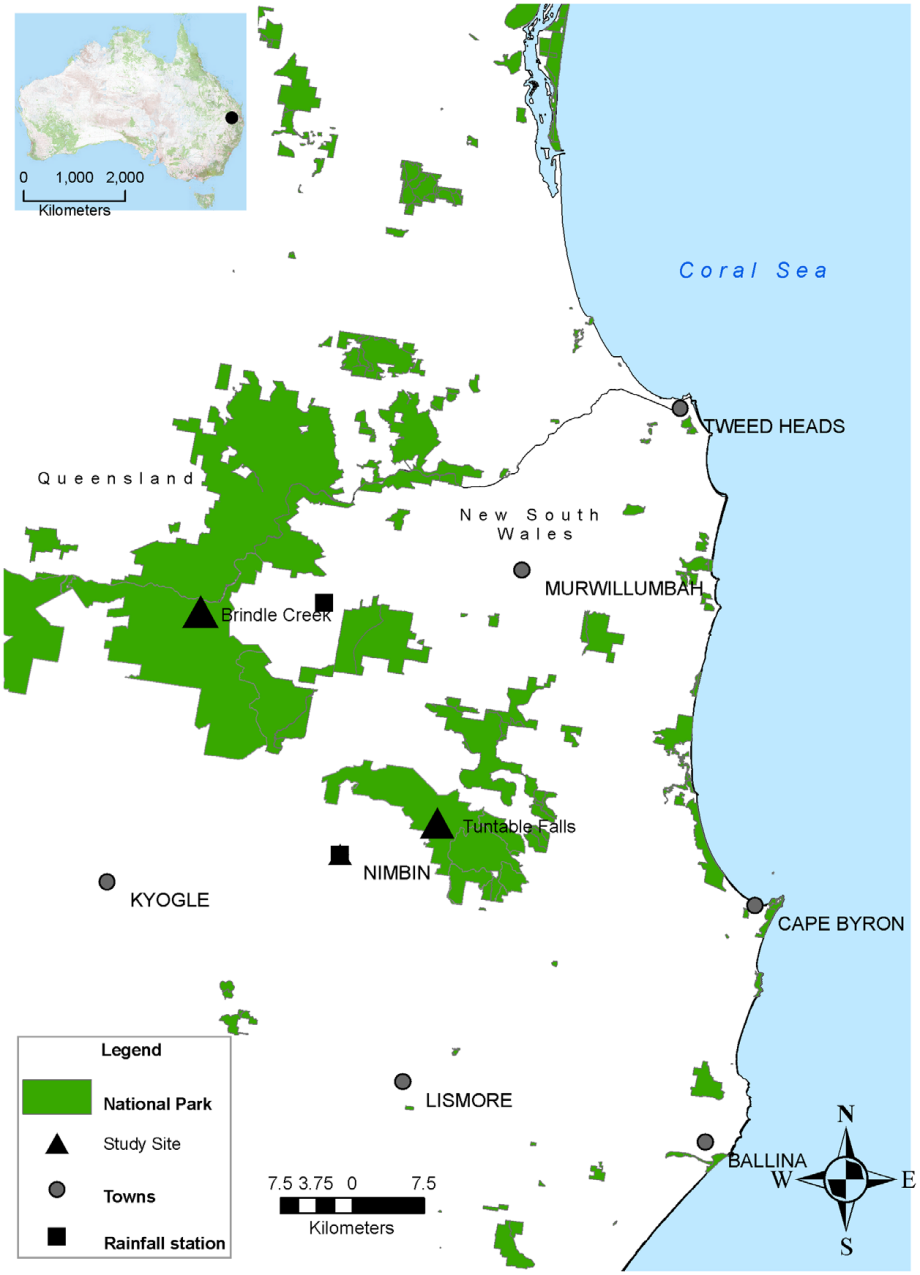
Location of the two study sites located approximately 30 km apart at Brindle Creek (Border Ranges National Park) and Tuntable Falls (Nightcap National Park).

This study was undertaken at two permanently flowing rainforest streams located approximately 30 km apart in northern New South Wales, Australia: Brindle Creek in Border Ranges National Park (elevation of 750 m) and Tuntable Falls in Nightcap National Park (elevation of 460 m) (Figure 1). Previous surveys [25] had identified remnant populations at these sites in what was considered to be high quality habitat. Both reserves are part of the World Heritage listed Gondwana Rainforest Reserves.

### Frog Survey and Marking

Surveys were conducted between November 2000 and March 2008. *Mixophyes fleayi* is intermittently active along streams between August and March (austral Spring – Autumn). A fixed transect of 100 m at Tuntable Falls and 200 m at Brindle Ck was searched at night (between approx. 2000–0400 h), by slowly traversing the stream and stream bank with the aid of a head torch and 50 W spotlight. Active frogs were detected via their eye-shine. *Mixophyes fleayi* is the only large frog present at Brindle Creek and is sympatric with *M. fasciolatus* at Tuntable Falls. Call playback and/or call mimicry were used at 10–20 m intervals along the stream in order to elicit a response from sheltering males. We recorded the temperature at the start of the survey and noted rainfall intensity during surveys. Additional rainfall data were obtained from the Bureau of Meteorology for the seven days prior to and after a survey from the two stations closest to our survey sites (see Figure 1).

Frogs were caught by hand using a plastic bag. Adult and sub-adult frogs (>40 mm) were tagged with a Passive Integrated Transponder (PIT) tag (Trovan Technologies) that was injected subcutaneously in the dorsum and read with the aid of a portable scanner. The site of injection was then sealed with Vetbond (®). A subset of frogs (65 individuals) was also toe clipped in order to assess the likelihood of tag loss. There was no evidence of tag loss or tag failure during the study.

### Data Analysis

Data were analysed using Mark [29] to estimate the probabilities of detection and adult survival, and to describe changes in population size over time. The analysis was focused on adult males because relatively few females were captured and only infrequently. The number of censuses varied between sites and across years. Between 4 and 51 censuses (139 in total) were conducted throughout the frog’s active period (August – March) under a variety of climatic conditions. Tuntable Falls was difficult to access during extended periods of wet weather. Censuses were not evenly spaced in time and were more frequent earlier than later in the series. Consequently, data were pooled into 12 occasions for Tuntable Falls and 14 occasions for the Brindle Creek, keeping periods with long intervals between them separate. The intervals of time between these pooled occasions were calculated as the difference in days between the average dates of pooled periods.

We constructed covariates to model capture probability as a function of effort and rainfall events. We considered that rainfall was likely to influence frog activity and that increased effort would result in a greater number of captures. Two alternative effort covariates were calculated: ‘effort’ as the number of individual surveys pooled into each occasion and ‘exp-effort’ as 1–(1–p*)*^t^* where p* was the estimated capture probability fitted as constant in a Cormack-Jolly-Seber (CJS) model using all of the individual survey data with time-varying survival (*φ*) and *t* was the number of individual surveys pooled into each sample.

Five alternative rain event covariates were calculated: ‘r7’, the proportion of individual surveys in an occasion that had rainfall in the previous 7 days; ‘sr7’, the proportion of individual surveys in an occasion that had substantial rain (>40 mm) in the previous 7 days; ‘r3’, the proportion of individual surveys in an occasion that had rain in the past 3 days; ‘sr3’, the proportion of individual surveys in an occasion that had substantial rain (>40 mm) in the past 3 days; and ‘r24’, the proportion of individual surveys in an occasion that had rain during the survey.

We used the POPAN formulation [30] of the Jolly-Seber (JS) model [31], [32] to generate apparent survival (φ**)** and capture probability (*p*) estimates and to examine population parameters. The POPAN model postulates the existence of a ‘super-population’ (*N*) consisting of the total number of animals that enter the study sites over the survey period and survive until the next sample time *i*. We estimated: apparent survival (permanent emigration and mortality cannot be distinguished), the probability of capture at each sample time and the probability of entry to the population. Further parameters were derived from these including: net recruits, the number of animals that enter after sample time *i* and survive to sample time *i*+1; and *N_i_*, the population size at sample time *i* (*i = *1….,*k*).

The POPAN parameterisation of the JS model assumes that: i) animals retain their tags throughout the study and tags are not misread; ii) sampling is instantaneous; iii) survival rates are the same for all animals (marked and unmarked) between each pair of sampling occasions (homogeneous survival); iv) catchablity is the same for all animals (marked and unmarked) at each sampling occasion; and v) the study area is constant.

### Model Selection

Model selection was based on Akaike’s information criterion with small sample correction (AICc), with the best fitting model indicated by lower AICc values, larger Akaike weights and larger model likelihoods [33]. Models were developed with combinations of time-varying and constant survival, capture and entry probabilities and with the effort and rain covariates on capture probabilities. For each site, the 10 models with the lowest AICc were selected for comparison.

We examined goodness of fit of the model to the data using program RELEASE (Test 2 & Test 3; [34]. In general terms, RELEASE tests the first assumptions of the model (equal catchability and survival) and departure of the data from the underlying assumptions of the model is indicated by the variance inflation factor (c-hat) and the associated *P* values from the chi-square (*x* ^2^) statistic. These tests use sample data to investigate the mathematical structure of the distribution [35].

### Ethics Statement

This research was conducted under permits issued by the NSW Department of Environment & Climate Change (A2895) and was approved by the Southern Cross University Animal Care and Ethics Committee (approval number 31/01) in accordance with the Australian Government National Health and Medical Research Councils Code of Practice for the Care and Use of Animals for Scientific Purposes.

## Results

We tagged 136 individuals (21 females) at Brindle Creek and 152 individuals (9 females) at Tuntable Falls. Our sampling encountered these individuals 974 times.

Goodness of fit tests in program RELEASE (Tests 2+3) indicated that the fully time dependent global model was an acceptable starting model for both Tuntable Falls (*x* ^2^ = 22.89, df = 20, *p* = 0.294, c-hat = 1.14) and Brindle Creek (*x* ^2^ = 24.14, df = 29, *p* = 0.722, c-hat = 0.83). Given the non-significant *p* values for the *x* ^2^ statistic and c-hat values close to 1, we did not adjust the variance inflation factor (c-hat).

### The Models

For both sites, the model with the lowest AICc had the same structure (Table 1). That is, apparent survival probability is constant over time with time varying probabilities of capture and entry. The likelihoods for this model were approximately three times as large as for the models with the next lowest AICc value. In contrast, models with entry probabilities constant over time had higher AICc values than models with time-varying survival probabilities. The AICc for the model phi(.)p(t)pent(.) was larger in each case than for the model phi(.)p(t)pent(t) (Δ AICc = 2.593 for Tuntable Falls and Δ AICc = 2.123 for Brindle Creek) indicating considerable support for models with time varying entry probabilities.

**Table 1:** The candidate models from the POPAN analysis for estimation of apparent survival (Phi (φ)), recapture (*p*) rates and the probability of entrance (pent) for the two locations.

Model^a^	AICc^b^	Delta AICc	AICc Weights	Model Likelihood	Number of Parameters
***Tuntable Falls***					
phi(.)p(t)pent(t)	396.148	0.000	0.478	1.000	22
phi(.)p(expeffort)pent(t)	398.384	2.236	0.156	0.327	15
phi(.)p(expeffort+sr7)pent(t)	398.538	2.390	0.145	0.303	16
phi(.)p(t)pent(.)	398.741	2.593	0.131	0.273	14
phi(.)p(expeffort+r7)pent(t)	400.493	4.345	0.054	0.114	16
phi(.)p(effort)pent(t)	401.723	5.575	0.029	0.062	15
phi(t)p(t)pent(.)	406.117	9.969	0.003	0.007	22
phi(t)p(expeffort)pent(.)	406.740	10.592	0.002	0.005	14
phi(t)p(effort)pent(t)	408.725	12.577	0.001	0.002	24
phi(t)p(expeffort+sr7)pent(.)	409.042	12.894	0.001	0.002	15
***Brindle Creek***					
phi(.)p(t)pent(t)	623.761	0.000	0.692	1.000	26
phi(.)p(t)pent(.)	625.884	2.123	0.239	0.346	14
phi(.)p(expeffort+sr7)pent(t)	629.896	6.135	0.032	0.047	17
phi(t)p(t)pent(t)	631.906	8.145	0.012	0.017	34
phi(.)p(effort+sr7)pent(t)	632.164	8.403	0.010	0.015	17
phi(t)p(expeffort+sr7)pent(t)	632.757	8.996	0.008	0.011	26
phi(t)p(t)pent(.)	634.856	11.095	0.003	0.004	23
phi(t)p(effort+sr7)pent(t)	635.181	11.420	0.002	0.003	27
phi(t)p(effort)pent(t)	636.637	12.876	0.001	0.002	26
phi(.)p(effort)pent(t)	638.604	14.843	0.000	0.001	16

a (*t*) = fully time-specific variation, (.) = variation is constant, (expeffort) = 1–(1–p*)*^t^*, (effort) = the number of surveys pooled in an occasion, (sr7) = substantial rainfall (>40 mm) in the previous 7 days.

b AIC_c_ is the estimated Akaike’s Information Criterion, the lower AICc values indicate better fitting models.

In both cases, the best fitting effort covariate was ‘expeffort’ while ‘sr7’ was the best fitting rain covariate (Table 1). In neither case, however, were these variables either alone or in combination able to fully account for the time-variation in capture probabilities.

### Capture Probabilities

**Figure 2:**
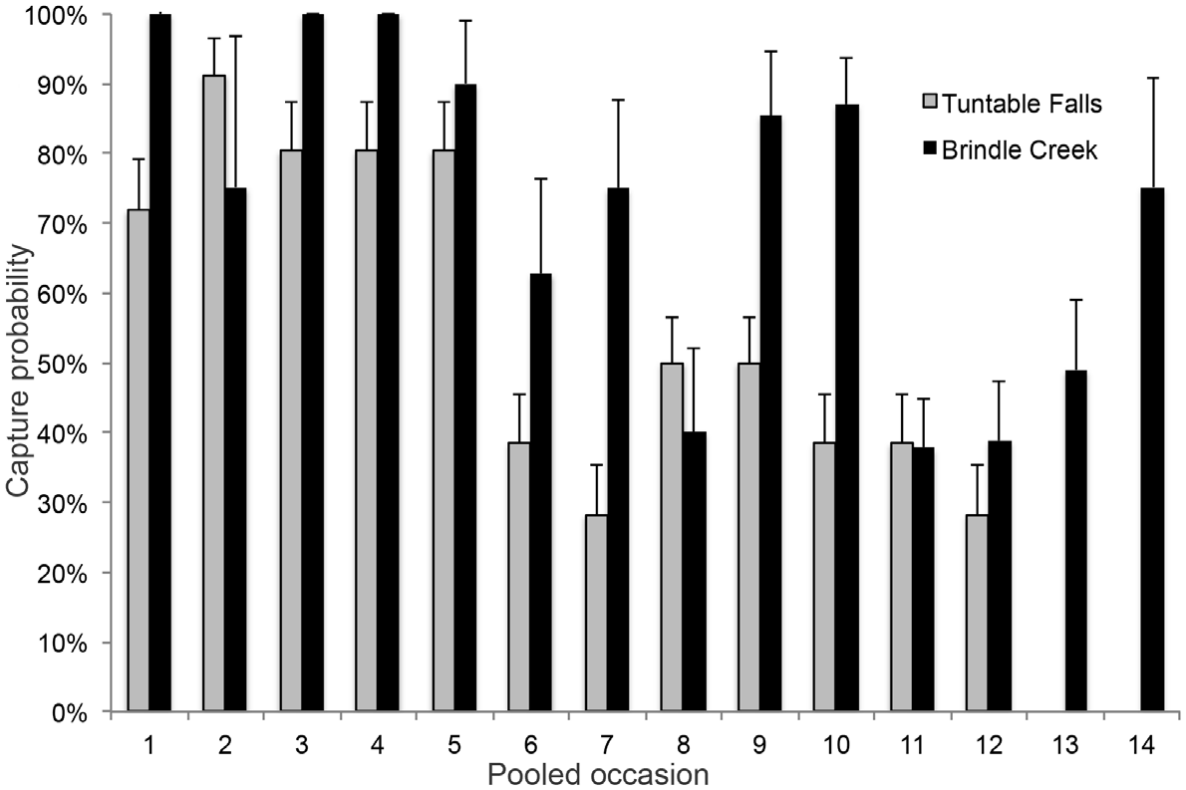
Estimates of capture probability (+SE) from the best fitting model (phi(.)p(t)pent(t)) for the two study sites.

Estimates of capture probability from the best fitting models were highly variable and ranged from 8% to 100% at Tuntable Falls and 38% to 100% at Brindle Creek (Figure 2). These estimates generally declined as the population increased and as the number of surveys decreased over time.

### Abundance (N) Estimation

**Figure 3:**
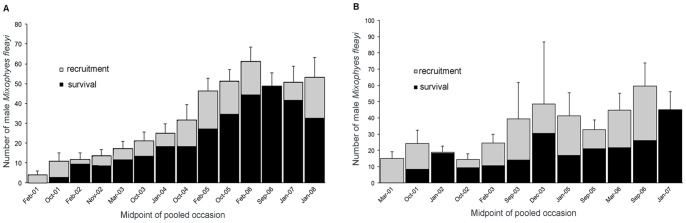
Derived population estimates (+SE) for male *M. fleayi* at our two study sites. A) Brindle Creek and B) Tuntable Falls and the relative contribution of recruitment and apparent survival. Dates are the average month for each pooled occasion used in the POPAN models.

The POPAN analysis was used to derive estimates of abundance based on the most supported model, namely phi(.)p(t)pent(t) (Table 1). The overall estimates of abundance of males (i.e.: the ‘super-population’) (N±SE) were 187 (±30) at Tuntable Falls to 135 (±7) for Brindle Creek. Abundance estimates increased from the start of the study at both sites but tended to stabilise at a mean of 40–60 frogs at each location during the last 3–4 years of the study (Figure 3). This represents a 3–10 fold increase in abundance over the study period.

### Survival Estimates

Surviving males comprised a large proportion of the population estimates at any time for both study sites (Figure 3). Apparent survival remained constant in the three best supported models at each site. The annual survival probability (±95% CI) at Brindle Creek (65%; U = 73%, L = 56%) is higher than the estimate for Tuntable Falls (38%; U = 48%, L = 28%).

The longest-lived male frog in the study had a capture history that spanned more than 6.4 years (2322 days) and was an adult at the time of first capture. This individual remained alive in the final occasion of the matrix. The longest-lived female had a capture history of 6.1 years.

### Recruitment Estimates

The probability of entry values ranged from 0–18% for Tuntable Falls and between 0–14% for Brindle Creek and was maintained at 4–6% at Brindle for occasions 3–6 (i.e. 2 yrs); then 10–14% for occasions 7–10 (i.e. 2 yrs). Net recruitment contributed slightly more to the population estimates at Tuntable Falls than at Brindle Creek (see Figure 3), however, no recruitment occurred at either site in the 11th interval. There were 12 pooled occasions at Tuntable Falls and thus only 11 intervals for that location.

## Discussion

### Population Increases Following Decline

We postulated that species that previously experienced *Bd*-induced declines may experience recurring periods of decline or increase in abundance over time. Our data demonstrate unambiguously that *M. fleayi* increased in abundance 3–10 fold over our study period at two independent sites. A rebound in population abundance following a period of decline has been observed in several other rainforest frogs but no prior study has demonstrated an unambiguous sustained recovery over a prolonged (>5 yr) period of time. In north-east Australia, the Eungella torrent frog (*Taudactylus eungellensis*) suffered a severe decline during 1985–6 but persisted at a few locations [10]. Mark-recapture analysis at these locations in the period 1994–98 revealed high survival rates (>0.5) in males and females, but no population estimates were presented. Further north the green-eyed frog (*Litoria genimaculata*) showed a severe decline in the period 1990–1993 but then a recovery during 1994–5 [22]. Mark-recapture analysis was required to demonstrate the change in abundance due to highly variable seasonal activity that may otherwise mask longer-term patterns. However, surveys did not extend for a sufficient period to unambiguously demonstrate that final abundance was stable at pre-decline levels. In Venezuela, the harlequin frog (*Atelopus cruciger*) had undergone a widespread decline and severe range contraction in 1986. Mark-recapture analysis showed that one remnant population was stable in the period 2005–07 [36]. Apparent recoveries such as these can only be demonstrated by detailed mark-recapture analysis because simple count data can be highly variable and do not take into account the important parameters of detectability and survival [37]. Understanding the on-going fate of amphibian populations in which *Bd* was implicated in population declines is fundamental to devising strategies for amphibian conservation.

### Survival and Detection Probabilities

In the absence of recruitment, either through immigration or successful breeding events, populations will eventually decline to extinction. Alford and Richards [38] postulated that due to the vagaries of juvenile recruitment, amphibian population dynamics may be characterized by declines. A key premise of this model is that amphibian populations are regulated by recruitment rather than by adult survival. Whilst this may be the case for some species, particularly those that inhabit highly seasonal habitats [39] and are short-lived (e.g. [36]), the situation is likely to differ between species and habitats. Stability in our *M. fleayi* populations appears to have been mediated by adult survival because juvenile recruitment was low. Biek *et al*. [40] demonstrated the importance of adult and juvenile survival rates in regulating population growth for three declining species using ecological sensitivity analysis. Apparent survival is a product of true demographic survival and site fidelity [35]. The estimates from Brindle Creek are likely to be closer to true demographic survival than those from Tuntable Falls where a shorter transect length allowed a greater opportunity for tagged frogs to move out of the study area. Our estimates of apparent annual survival of adult male frogs were stable and sufficiently high at both sites (Brindle: 65%; Tuntable: 38%) for populations to increase in light of low recruitment.

Indicative of high survivorship was the presence of long-lived individuals. One male, an adult at first capture, was present for at least 6.4 years. A female, first tagged as an adult, was detected in the last capture session after 6.1 years. Long-lived amphibians may be more able to recover from periods of decline than those that are short-lived [41] because reproductive failure between consecutive years may be of limited consequence to their extinction probability. Whilst this may lead to shifts in population age structure, factors such as the connectedness of breeding habitats and the dispersal ability of species (in the absence of catastrophic events) will ultimately influence the probability of extinction. Being long-lived has some important implications for the conservation of *M. fleayi*. Firstly, impacts on reproductive output may take many years to be recognised as a decline in the number of adult frogs at a site and surveys based on counts of calling males alone would fail to detect this for many years. Conversely, sudden changes in adult survival may result in precipitous declines and may account for the sudden decline of this species in the mid-1990s.

One factor that commonly confounds studies of frog population dynamics is the probability of detection [42]. Our modeling revealed that this probability was above 50% for *M. fleayi* males for the majority of sampling occasions at both locations and was mostly above 70% during the first two years. High detection probability adds weight to previous inferences of rarity that were based on counts alone [8], [25]. Detection probability was more variable with increasing population size and decreased survey effort in the latter stages of our study. Our results are in stark contrast to the 7-year study on *L. genimaculata*, in which most frogs were captured only once [22] most likely because these frogs regularly dispersed to and from the surrounding forest [22], [43].

### Male Demography

One obvious limitation of our study is that our analysis is based on the male component of the two populations. As with most studies of amphibians, sampling was associated with breeding habitat and although extensive areas of adjacent forest were regularly traversed, few females were ever detected. Such limitations may be a common feature of studies that rely on sampling at breeding sites [22], [38], [44], [45], particularly when the methods are biased towards detection of calling males. The question is whether this precludes us from reaching a conclusion about the broader population dynamics of *M. fleayi*. For both our populations, the number of adult males increased in most years and survivorship values were high. It seems unlikely that these populations could increase in size if only the male segment of the population was increasing. Sex-specific differences in capture probabilities of frogs is reasonably widespread (e.g. [46]). We postulate that the female component of the population would follow a trajectory correlated with that of the male component regardless of the operational sex ratios.

### Management Implications

This study is one of the few to conduct a detailed mark-recapture analysis of frog populations in the rainforests of eastern Australia. Given that this geographic area has been a focal point for frog declines in the past, there is a need to conduct similar studies over longer time periods. Populations of *M. fleayi* may still be vulnerable to future declines, or may be adversely affected by climate change. We have demonstrated that this species has a high probability of detection so that simple transect counts may be adequate to describe the dynamics of populations. Monitoring could be extended to other locations to provide a robust account of the population dynamics of this species more broadly (e.g. [47]) and their ability to persist in light of *Bd*.

Determining the relative contributions of recruitment and adult survival to overall population growth has important implications for the focus of future conservation and research effort. Ultimately, adult survival will determine the length of time a population may persist without successful recruitment (e.g. [48], [49]). Efforts focused on increasing survival of eggs or larvae in aquatic habitats may not be appropriate if adult survival in terrestrial habitats is more important to overall population size. Biek *et al.* [40] showed that changes in adult or juvenile survival can be more important in governing population growth than embryonic or larval survival. In the case of *M. fleayi* it appears that efforts focused on terrestrial habitats will be important as adult survival contributed substantially to population persistence. Whilst adult survival has allowed populations to persist, ultimately successful breeding and recruitment leads to population growth. Longevity may be central in buffering populations from periods of low recruitment.

*Bd* varies in its impact across species and habitats, even in cases where potential hosts are sympatric [1], [38]. McDonald *et al.* [14] suggested that *Bd* has now become endemic in north Queensland frog populations and that infection prevalence had declined. They noted that detection of *Bd* was significantly associated with season and altitude (greatest above 300 m during winter) and suggest that one species, *L. genimaculata,* had increased in abundance to pre-decline numbers, despite moderate prevalence rates (7.8%). Further research is required to determine if a shift in the host pathogen relationship has occurred. This would apply to several species including *M. fleayi*. Such shifts may be mediated either via changes in environmental conditions, decreased transmission and virulence, or the development of resistance and acquired immunity [50].
